# The Effect of Upright Stance and Vision on a Cognitive Task in Elderly Subjects and Patients with Parkinson’s Disease

**DOI:** 10.3390/brainsci14040305

**Published:** 2024-03-24

**Authors:** Marta Mirando, Rachele Penati, Marco Godi, Marica Giardini, Antonio Nardone

**Affiliations:** 1 Department of Clinical-Surgical, Diagnostic and Pediatric Sciences, University of Pavia, 27100 Pavia, Italy; marta.mirando@icsmaugeri.it (M.M.); rpenati@valduce.it (R.P.); 2 Physical Medicine and Rehabilitation Unit of Veruno Institute, Istituti Clinici Scientifici Maugeri IRCCS, 28010 Veruno, Italy; marco.godi@icsmaugeri.it (M.G.); marica.giardini@icsmaugeri.it (M.G.); 3 Centro Studi Attività Motorie and Neurorehabilitation and Spinal Units of Pavia Institute, Istituti Clinici Scientifici Maugeri IRCCS, 27100 Pavia, Italy

**Keywords:** posture, balance, dual task, serial subtraction, Parkinson’s disease, rehabilitation

## Abstract

Standing compared to sitting enhances cognitive performance in healthy subjects. The effect of stance on cognitive performance has been addressed here in patients with Parkinson’s disease (PwPD). We hypothesized that a simple cognitive task would be less enhanced in PwPD by standing with respect to sitting, because of a larger cognitive effort for maintenance of standing posture than in healthy subjects. We recruited 40 subjects (20 PwPD and 20 age-matched healthy subjects, HE). Each participant performed an arithmetic task (backward counting aloud by 7) in two postural states, sitting and standing, with eyes open (EO) and with eyes closed (EC). All trials lasted 60 s and were randomized across subjects and conditions. The number of correct subtractions per trial was an index of counting efficiency and the ratio of correct subtractions to total subtractions was an index of accuracy. All conditions collapsed, the efficiency of the cognitive task was significantly lower in PwPD than HE, whilst accuracy was affected to a lower extent. Efficiency significantly improved from sitting to standing in HE under both visual conditions whilst only with EO in PwPD. Accuracy was not affected by posture or vision in either group. We suggest that standing, compared to sitting, increases arousal, thus improving the cognitive performance in HE. Conversely, in PwPD this improvement was present only with vision, possibly due to their greater balance impairment with EC consuming an excess of attentional resources. These findings have implications for balance control and the risk of falling in PwPD in the absence of visual cues.

## 1. Introduction

Although quiet stance might be considered a simple motor task [1], it entails the utilization of both subcortical and cortical brain areas, which integrate the different visual, somatosensory, and vestibular inputs (see [2], for a review). Even the simple act of standing leads to a modulation of the early components of somatosensory cortical potentials. This has been interpreted as the descending modulation of excitability of spinal circuits engaged in reflex balance control [3,4].

In addition to the appropriate reflex modulation, stance requires attention [5,6,7], the amount of which depends on the difficulty of the postural task [8]. For instance, standing on one foot or a narrow support base requires a higher amount of cognitive resources compared to a bipedal stance, in both adolescents and young adults [9]. On the other hand, an upright stance, compared to sitting, increases cognitive performance as if standing mobilizes cognitive resources thereby favoring a general enhancement of the cognitive performance. This has been proven in young subjects using the Stroop test [10,11], and has been interpreted as an effect of enhanced attentional selectivity to the environment during standing [10].

Elderly subjects show reduced speed of cognitive information processing when compared to young subjects. This impairment has been attributed to loss of brain integrity, particularly in the frontal and parietal lobes [12]. Further slowing in cognitive information processing has been shown in PwPD [13] and attributed to loss of white matter connected to small vessel disease (see [14] for a recent review). In these patients, impairments in cortico-basal ganglia circuits and cholinergic systems [15] often lead to impaired working memory, independently of dopaminergic medication [16,17,18].

Standing upright, compared to sitting, increases cognitive performance in young subjects [10,11] and cognitive information processing is more impaired in PwPD than in elderly subjects [13]. Therefore, we assumed that the standing posture with respect to sitting might be advantageous in performing an easy cognitive task in elderly subjects but not in PwPD, considering their difficulties in balance control during standing [19].

To our knowledge, the studies addressing this issue are those by Sciadas et al. [20] and Morenilla et al. [21]. Sciadas et al. [20] found a decrease in the cognitive performance of a verbal naming task in both elderly subjects and PwPD when standing compared to a sitting posture. Morenilla et al. [21] compared cognitive performance consisting of a phoneme monitoring task during sitting and standing as still as possible in elderly subjects and PwPD. They found an improvement in the cognitive performance of elderly subjects when standing compared to sitting with eyes open but not with eyes closed. On the contrary, the cognitive performance of PwPD diminished when standing compared to sitting but the effect occurred only with eyes open. The different and partly contrasting results obtained in the above two studies might be connected to the different cognitive tasks and postural requirements. Therefore, in the present study, we reevaluated the cognitive performance of elderly subjects and PwPD on a simple serial subtraction task under sitting and standing conditions. We asked whether cognitive performance improves to a similar extent in elderly subjects and PwPD with standing since any positive posture–cognitive interaction might be exploited in future diagnostic approaches (see [22], for a recent review) or rehabilitation programs [23]. We instructed subjects to maintain a quiet rather than still upright posture to reduce conscious control of the upright stance [24], thus releasing cognitive resources for the subtraction task and avoiding an unduly worsening of cognitive performance [20]. In addition, we took into consideration the influence of vision on cognitive performance under the two postural conditions. In fact, it has been shown that body sway increases in PwPD with respect to elderly subjects when required to perform a simple arithmetic task while standing [25]. This instability might be counteracted by possibly diverting more cognitive resources to the postural task compared to elderly subjects.

## 2. Materials and Methods

Patients with Parkinson’s disease (PwPD) were referred from the Physical Medicine and Rehabilitation Unit of Istituti Clinici Scientifici Maugeri Spa SB (IRCCS) of Veruno (Novara, Italy). Healthy elderly subjects (HE) were spouses or companions of the PwPD. The following inclusion criteria were applied to both groups: (1) age > 60 years old; (2) absence of central and peripheral nervous system diseases except for PD; (3) no major trauma or orthopedic surgical intervention in the last 6 months as obtained from the clinical history; (4) Mini-Mental State Examination (MMSE) score ≥ 23 (corrected for age and educational level) [26]. The MMSE was administered in a non-blinded fashion.

We enrolled a total of 40 subjects, of which 20 were HE and 20 PwPD (see Table 1). All PwPD were on stable medication and no patient assumed anticholinergic drugs suspected to affect cognitive decline [27]. They were “on phase” at the time of the evaluation.

The study was approved (#651/2010) by the Ethics Committee of the Istituti Clinici Scientifici Maugeri of Pavia and signed informed consent was obtained from each subject and patient before participation.

Our study was powered to investigate the impact of two distinct postural conditions (sitting vs. standing) on cognitive task performance. To determine the appropriate sample size, we conducted an a priori calculation based on data from Morenilla et al. [21], which explored changes in cognitive task performance during upright stance in both PwPD and HE. Their findings revealed a decrease in cognitive performance, as assessed with a phoneme monitoring task, in PwPD during standing compared to HE. The observed difference in cognitive performance between the groups corresponded to an effect size (Cohen’s d) of approximately 1. Therefore, a minimum sample size of 20 participants per group (HE; PwPD) was determined to achieve a statistical power of 90%, with a significance level set at 0.05.

The arithmetic task consisted of backward counting aloud by 7, starting from a random number between 100 and 130. The task was performed under two postures: sitting on a comfortable chair with a backrest and standing at ease barefoot with the feet at shoulder width, a distance allowing stable posture [19]. Both postures were maintained with eyes open (EO) gazing at the black center (10 mm diameter) of a shooting target (30 cm diameter) placed at a distance of 50 cm at eye level, and with eyes closed (EC). Each arithmetic task was set to 60 s and the order of all trials (sitting or standing, and with EO or EC) was randomized for each participant. Within the randomization sequence, each test condition was repeated two times for a total of eight trials. No cue was given to the subjects regarding prioritization of balance control during standing or performance of the arithmetic task. Between trials, participants were allowed to sit when necessary. The entire experiment was performed within 30 min.

Within each counting sequence, we recorded the number of total subtractions that included both the correct subtractions and the errors. From these data, we separately calculated the number of correct subtractions during the 60-s trial and the ratio of the number of correct subtractions to the number of total subtractions, i.e., efficiency and accuracy of the cognitive performance, respectively [28].

For each subject, the efficiency and accuracy of the cognitive task were calculated from the mean values of the two trials obtained under the same postural and visual conditions. Consequently, each subject entered the database with four values of efficiency and accuracy corresponding to the following conditions: sitting EO, standing EO, sitting EC, and standing EC.

In the tables and figures, when not differently stated, the data are presented as means ± standard error (SE). For the differences in the clinical characteristics of the participants, the Chi-squared or the unpaired Student’s *t*-test were used when relevant. A 3-way ANOVA between groups (HE and PwPD) as independent factors and within two repeated measures (sitting and standing, EO and EC) was separately performed on efficiency and accuracy values of the cognitive performance. *p*-values lower than 0.05 were considered significant. To allow correction for multiple comparisons, a post hoc analysis was performed with Tukey’s HSD test. All statistical tests were performed using the software Statistica^®^ version 7 (Tulsa, OK, USA).

## 3. Results

### 3.1. Participant Characteristics

Table 1 summarizes the clinical characteristics of the participants. We found no significant difference in sex distribution (Chi-squared test, *p* = 0.75) between HE and PwPD. Mean differences in age and Mini-Mental State Examination (MMSE) scores between the two groups were also not significant (unpaired Student’s *t*-test, respectively, *p* = 0.13 and *p* = 0.82). The Hoehn–Yahr score ranged from 1.5 to 3 and the UPDRS score from 8 to 51. Duration of the disease averaged 7.1 years, ranging from 1 to 15 years. Accordingly, the levodopa equivalent daily dose (LEDD) ranged from 188 to 1188 mg.

### 3.2. Efficiency of the Cognitive Performance under the Different Postural and Visual Conditions

**Figure 1:**
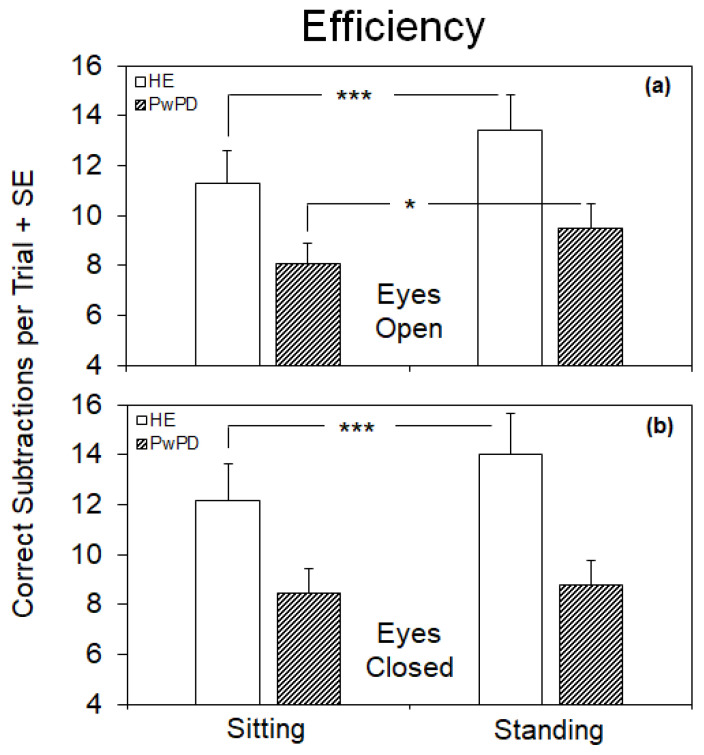
Mean values (+standard error, SE) of the efficiency (number of correct subtractions during the 60-s trial) of the arithmetic task in healthy elderly (HE) subjects and patients with Parkinson’s disease (PwPD) under sitting and standing conditions with eyes open (**a**) and eyes closed (**b**). With eyes open, in both HE and PwPD efficiency was higher on standing than sitting, whilst with eyes closed efficiency increased only in HE. *, *p* < 0.05; ***, *p* < 0.001.

Table 2 and Figure 1 show that the mean efficiency (i.e., the number of correct subtractions during the 60-s trial) of the arithmetic task was significantly higher (ANOVA, *p* = 0.02) in HE compared to PwPD. In addition, an effect of postural condition was observed (ANOVA, *p* = 0.004), since mean efficiency increased under standing with respect to sitting conditions. This effect was evident under both visual conditions in HE whilst only with EO in PwPD. In fact, with EO, passing from sitting to standing, efficiency significantly increased in both HE and PwPD (post hoc, *p* < 0.0005 and *p* < 0.01, respectively). On the other hand, with EC, efficiency significantly increased only in HE (post hoc, *p* = 0.001). 

### 3.3. Accuracy of the Cognitive Performance under the Different Postural and Visual Conditions

**Figure 2:**
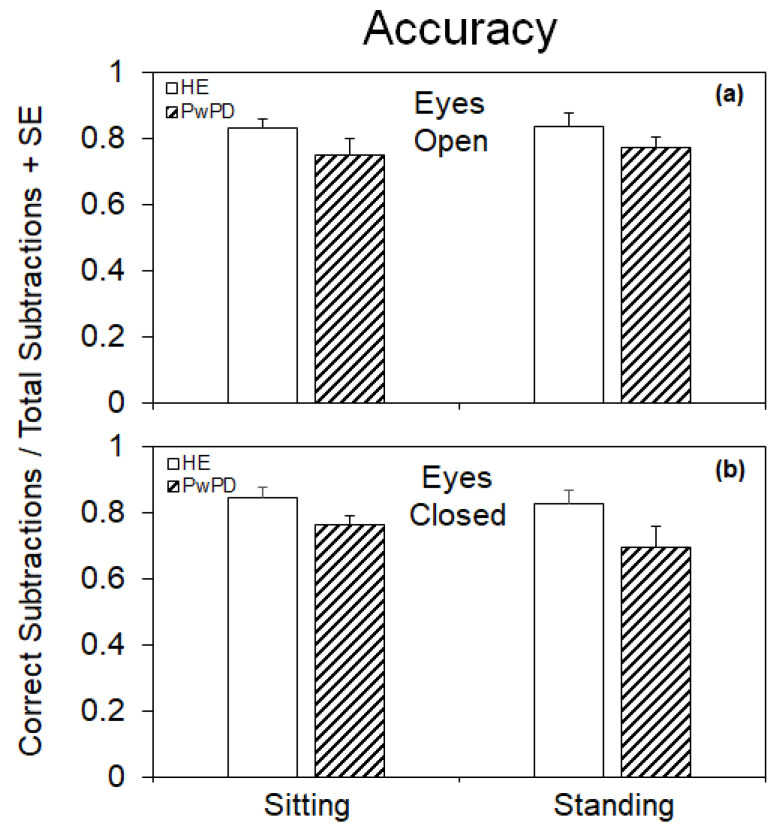
Mean values (+standard error, SE) of the accuracy (ratio of the number of correct subtractions to the number of total subtractions) of the cognitive performance task in healthy elderly (HE) subjects and patients with Parkinson’s disease (PwPD) under sitting and standing conditions with eyes open (**a**) and eyes closed (**b**). Accuracy was only marginally significantly smaller in PwPD than HE under all postural and visual conditions. Standing did not improve accuracy in either group.

Table 3 and Figure 2 show that the mean accuracy (i.e., the ratio of the number of correct subtractions to the number of total subtractions) of the arithmetic task was only marginally (ANOVA, *p* = 0.08) smaller in PwPD compared to HE. All other main effects and interactions were not significant. Hence, overall, accuracy was hardly affected by posture or vision in either subject group.

### 3.4. Relationship between Cognitive Performance under the Different Postural and Visual Conditions and the Clinical Findings

As expected, in PwPD, the levodopa equivalent daily dose (LEDD) was significantly (y = 49.3x + 319.3, *p* = 0.001, R^2^ = 0.45) higher as a function of the duration of the disease. However, neither the efficiency nor accuracy of the cognitive performance nor the MMSE score were affected by the duration of the disease or LEDD. Finally, no relationship was found between efficiency or accuracy values and the Hoehn–Yahr stage or UPDRS score.

## 4. Discussion

We assessed the differential effect of two postural tasks (sitting vs. standing) on a simple cognitive task (backward counting) in patients with Parkinson’s Disease (PwPD) compared to age-matched healthy elderlies (HE). In addition, the influence of vision on cognitive performance was assessed under the two postural conditions. The effect of the two postural and visual conditions on cognitive performance was measured by recording the number of total subtractions and the correct subtractions [29]. From these data, we separately measured the number of correct subtractions during the 60-s trial duration (i.e., the efficiency of the cognitive performance) and the ratio of the number of correct subtractions to the number of total subtractions (i.e., the accuracy of the performance) [28].

### 4.1. Differences between Efficiency and Accuracy of the Cognitive Task under the Different Postural and Visual Conditions in HE

We found that in HE the cognitive performance estimated with the efficiency increased from sitting to standing under both visual conditions. In fact, the number of correct subtractions increased within the 60-s trial duration. However, there was no increase in the absolute number of correct responses with respect to the total number of subtractions because accuracy did not increase. Therefore, the improvement in the arithmetic task under standing conditions was not connected with an improved ability of calculation. On the contrary, it is conceivable that standing upright favored an increase in the calculation rhythm and, in turn, in the number of correct subtractions. The advantage observed in efficiency during the arithmetic task while standing in HE might be connected to the fact that an upright stance leads to an “arousal effect” [10]. An enhancement of attentional selectivity to the environment and of cognitive control during standing compared to sitting has also been recently demonstrated in healthy young adults [10]. This is in keeping with the hypothesis that standing, a moderately demanding task, may recruit additional cognitive resources useful for a concomitant cognitive task. This enhancement of cognitive performance during standing might be helpful during daily life since an upright rather than seated posture allows better control of the environment and identification of possible hazards [10].

### 4.2. Visual Deprivation Hampers the Improvement of the Cognitive Task While Standing in PwPD

Also, in PwPD efficiency of the cognitive performance improved during upright posture but this was observed only with eyes open (EO) whilst it was negligible with eyes closed (EC). It is, therefore, plausible that with EO PwPD are capable of taking advantage of the “arousal effect” connected with the upright position as much as HE. However, also in the case of PwPD, accuracy did not improve. This means that the increase in number of correct subtractions with EO was an effect related to the increase in the total number of subtractions within the 60-s trials rather than to an improved calculation ability.

The negligible improvement in efficiency from sitting to standing with EC might be due to the already described worsening of balance control of PwPD during a simultaneous cognitive task similar to ours [19]. This worsening might consume more attentional resources for balance control or induce anxiety [30], and these effects might be stronger during standing with EC than EO [20]. As a consequence, this effort to maintain balance with EC would affect the performance of a simultaneous cognitive task [20]. The effort during standing might be a consequence of the prioritization of postural control over the concurrent cognitive task [31,32] with EC, a phenomenon present in PwPD, likely due to the perception of an augmented risk of fall. In other words, standing upright with EC might represent a more demanding task in PwPD than in HE, thus hampering the advantage of an upright stance in enhancing attentional resources observed in HE. On the contrary, the relatively little challenge of the standing task associated with normal balance control in HE would allow them to take advantage of an upright stance to improve cognitive performance [10] regardless of vision conditions.

We are aware that the smaller improvement of the cognitive task during standing with EC in PwPD might be attributed to the instruction given for posture maintenance. In fact, as shown by Sciadas et al. [20], requiring PwPD to stand still worsens cognitive performance. This explanation would not hold true for our case since subjects were instructed to stand at ease rather than as still as possible. It is, however, possible that a sort of involuntary “rigid” postural attitude [33] might be present in these patients, particularly during stance with EC, thus negatively interfering with the cognitive task [24].

The negligible improvement of cognitive performance in PwPD during standing with EC occurred even if they were in “on state” of dopaminergic medications and had only mild to moderate motor impairment. Of note, the difficulty in improving cognitive performance did not depend on cognitive impairment since the mean MMSE of PwPD was similar to that of HE. This difficulty is not completely unexpected since it is known that dopaminergic medication improves motor symptoms but worsens balance control [34] and bears controversial effects on cognitive functions [35]. Not unexpectedly, in our sample of not severely affected PwPD we did not find a relationship between efficiency (or accuracy) of the cognitive performance, or MMSE, and levodopa equivalent daily dose. Indeed, both positive and negative effects of dopaminergic medication have been described and it has been suggested that these effects depend on task demands [35].

At variance with the study by Abou-Khalil et al. [36], showing that standing does not affect a serial subtraction task with respect to sitting, we found a moderate improvement of the cognitive task under standing with respect to sitting posture in HE under both visual conditions and only with EO in PwPD. This difference with our study might be related to the fact that in the study by Abou-Khalil et al. [36], the sample was represented by young subjects rather than HE or PwPD. Therefore, it is conceivable that the cognitive resources of young subjects are at their best to be a little increasable by changes in posture, a sort of “ceiling” effect. On the contrary, our samples were composed of HE and PwPD whose cognitive resources are known to be reduced with respect to those of young subjects [12]. Therefore, paradoxically, the reduced cognitive status of HE and PwPD might have allowed them to take advantage of the “arousal effect”, thus improving the performance of the arithmetic task when passing from sitting to standing.

One possible neural structure involved in the enhancement of cognitive performance under standing conditions might be the prefrontal cortex which is known to play a role not only in executive functions [37], but also to be activated in upright posture [38]. The prefrontal cortex might be also involved in the inability of PwPD to reach the same efficiency of cognitive performance of HE. In fact, on the one hand, cognitive dysfunction in PD has been also connected to abnormal processes that localize to the prefrontal dopaminergic circuit [39]. On the other hand, this circuit also plays a role in the balance control of PwPD as suggested by the improvement of balance after transcranial direct current stimulation of the prefrontal cortex [40]. Therefore, it is conceivable that the prefrontal cortex of PwPD is less efficient than that of HE in managing the posture–cognitive interaction during the simultaneous performance of a cognitive task while standing, particularly with EC [41]. As an alternative interpretation, the reduced improvement of the efficiency of the cognitive task during standing might be connected to motor speech abnormalities like hypokinetic dysarthria [42]. However, this interpretation seems at odds with the finding that the increase in the number of total subtractions within the standing trial was present with EO.

Morenilla et al. [21] also compared the cognitive performance in HE and PwPD during sitting and standing conditions. In particular, they showed an improvement in the cognitive performance of HE when standing compared to sitting with EO but not with EC. On the other hand, PwPD even worsened cognitive performance when standing compared to sitting but the effect occurred only with EO. This finding seems to be unexpected since it is known that closing the eyes increases instability [43,44] that might be counteracted at the expense of engagement of further cognitive resources [20], thus affecting the performance of a simultaneous cognitive task in PwPD. At variance with the results by Morenilla et al. [21], in our study, the cognitive performance of HE improved under both visual conditions but this improvement was present only with EO in PwPD. We do not have a simple explanation for the contrasting results, but we hypothesize that the difference with our study might be in part connected to the different cognitive tasks required to the subjects. In fact, it is conceivable that in the phoneme monitoring task, a greater competition than in the arithmetic task occurs between balance control and cognitive performance. In addition, these authors required subjects to stand as still as possible rather than standing at ease as in our study. Standing as still as possible worsens cognitive performance since it increases attentional demands, per se, as already reported [20,24].

### 4.3. Relationship between Clinical Variables and the Cognitive Task

Although it is known that the disease itself [14] and/or the dopaminergic medication [35] can affect the cognitive capacity of PwPD, we did not find a dependence of the Mini-Mental State Examination (MMSE) scores on the duration of the disease or levodopa equivalent daily dose as well as on clinical evaluations performed with Hoehn–Yahr staging or UPDRS. This negative result might be connected with the fact that in our study, PwPD were only mildly to moderately affected by the disease [45]. It is conceivable that the reduced efficiency of PwPD should depend on a disease-related difficulty in the capability to perform the arithmetic task. This difficulty might be the epiphenomenon of cognitive slowing, which is independent of motor slowing and present also in cognitively unimpaired PwPD [46].

### 4.4. Limitations

We did not measure body sway. The main reason for this choice is that we were interested in the influence of sitting and standing conditions on cognitive performance during a simple arithmetic task rather than in the effects of the cognitive task on balance control itself [25]. In addition, the postural conditions (sitting vs. standing) might prevent comparable measurements of body sway [47]. Further, one has to consider that the body sway of PwPD during quiet stance shows only a slight increase with respect to HE [48] and that during the trials, an upright stance was maintained with the feet at shoulder width to increase posture stability [19] and to optimize the release of resources for the simultaneous cognitive task. These two conditions and the variability in disease duration and severity of PwPD could have reduced the sensitivity of the body sway evaluation in detecting changes between postural conditions.

## 5. Conclusions

It seems safe to conclude that in a standing condition with EC, a simple arithmetic subtraction task discloses a major allocation of attention in PwPD compared to sitting. On the contrary, HE take advantage of an upright stance under both visual conditions owing to their smaller allocation of attention to balance control [49]. These results can be interpreted in light of the prioritization of postural control over concurrent cognitive tasks in PwPD [31], a phenomenon already described during gait under difficult conditions such as when PwPD are required to walk toward and step over an illuminated obstacle in the dark [50]. In this context, it should be remembered that vestibular [51] and proprioceptive signal processing [52] are both impaired in PwPD, supporting previous findings of an increased visual dependence of these patients [53]. Our findings could thus be considered concerning the control of equilibrium and the risk of falling in PwPD, taking into account the impaired modulation of a simple cognitive task by vision or body position. On the other hand, it is conceivable that training the cognitive task during standing might improve both the postural task [54,55] and the cognitive performance in PwPD [23], as also already shown in healthy young adults [56]. This type of training seems to contribute to reducing fall risk in patients with neurological diseases during daily activities [57].

## Figures and Tables

**Table 1:** Clinical characteristics of the participants.

Characteristics	HE (*n* = 20)	PwPD (*n* = 20)	*p* Value
Sex	9 M, 11 W	10 M, 10 W	0.75 ^#^
Age (years)	68.5 ± 8.8	72.3 ± 2.1	0.13 ^†^
Disease duration (years)	n/a	7.1 ± 4.5	n/a
Hoehn-Yahr scale (score)	n/a	2.5 ± 0.5	n/a
UPDRS motor section (score)	n/a	24.8 ± 11.5	n/a
LEDD (mg)	n/a	667.1 ± 328.8	n/a
Mini-Mental State Examination (score)	27.3 ± 1.8	27.1 ± 2.1	0.82 ^†^

HE, healthy elderlies; PwPD, patients with Parkinson’s disease; M, men; W, women; UPDRS, Unified Parkinson’s Disease Rating Scale; LEDD, levodopa equivalent daily dose; n/a, not applicable. Means ± standard deviation. ^#^, Chi squared test; ^†^, unpaired Student’s *t*-test.

**Table 2:** Results of the analysis of variance performed on the efficiency (number of correct subtractions during the 60-s trial) of the arithmetic task under the two postural (sitting, standing) and visual (eyes open, eyes closed) conditions between the two subject groups.

Conditions	F	dF	*p*-Value
Group (HE-PwPD)	5.84	1.38	0.02
Posture (Sitting-Standing)	9.29	1.38	0.004
Vision (EO-EC)	0.98	1.38	0.33
Group × Posture	1.40	1.38	0.24
Group × Vision	2.59	1.38	0.12
Posture × Vision	3.95	1.38	0.05
Group × Posture × Vision	1.10	1.38	0.30

HE, healthy elderlies; PwPD, patients with Parkinson’s disease; EO, eyes open; EC, eyes closed; dF, degrees of freedom.

**Table 3:** Results of the analysis of variance performed on the accuracy (ratio of the number of correct subtractions to the number of total subtractions) of the arithmetic task under the two postural (sitting, standing) and visual (eyes open, eyes closed) conditions between the two subject groups.

Conditions	F	dF	*p*-Value
Group (HE-PwPD)	3.13	1.38	0.08
Posture (Sitting-Standing)	0.51	1.38	0.48
Vision (EO-EC)	1.84	1.38	0.18
Group × Posture	0.15	1.38	0.70
Group × Vision	2.25	1.38	0.14
Posture × Vision	1.26	1.38	0.27
Group × Posture × Vision	0.04	1.38	0.84

HE, healthy elderlies; PwPD, patients with Parkinson’s disease; EO, eyes open; EC, eyes closed; dF, degrees of freedom.

## Data Availability

The mean data presented in this study are available on request from the corresponding author. The dataset is not publicly available since the authors intend to use the dataset for future research and analyses.
